# Genetics and Biochemistry of Zero-Tannin Lentils

**DOI:** 10.1371/journal.pone.0164624

**Published:** 2016-10-27

**Authors:** Mahla Mirali, Randy W. Purves, Rob Stonehouse, Rui Song, Kirstin Bett, Albert Vandenberg

**Affiliations:** Plant Sciences Department, University of Saskatchewan, Saskatoon, SK, Canada; Henan Agricultural University, CHINA

## Abstract

The zero-tannin trait in lentil is controlled by a single recessive gene (*tan*) that results in a phenotype characterized by green stems, white flowers, and thin, transparent, or translucent seed coats. Genes that result in zero-tannin characteristics are useful for studies of seed coat pigmentation and biochemical characters because they have altered pigmentation. In this study, one of the major groups of plant pigments, phenolic compounds, was compared among zero-tannin and normal phenotypes and genotypes of lentil. Biochemical data were obtained by liquid chromatography-mass spectrometry (LC-MS). Genomic sequencing was used to identify a candidate gene for the *tan* locus. Phenolic compound profiling revealed that myricetin, dihydromyricetin, flavan-3-ols, and proanthocyanidins are only detected in normal lentil phenotypes and not in zero-tannin types. The molecular analysis showed that the *tan* gene encodes a bHLH transcription factor, homologous to the *A* gene in pea. The results of this study suggest that *tan* as a bHLH transcription factor interacts with the regulatory genes in the biochemical pathway of phenolic compounds starting from flavonoid-3’,5’-hydroxylase (*F3’5’H*) and dihydroflavonol reductase (*DFR*).

## Introduction

Phenolic compounds are characterized by the presence of at least one -OH group and an aromatic ring. They include phenolic acids, stilbenes, and flavonoids such as flavanones, flavones, dihydroflavonols, flavonols, flavan-3-ols, anthocyanidins, and proanthocyanidins [1]. Phenolics are associated with health benefits including antioxidant activity and protection against diseases such as cardiovascular disorders, cancer, HIV, and diabetes [2–6]. Physical removal of the seed coat of lentils leads to improved iron bioavailability [7], probably due to the removal of phenolic compounds and the implication that these compounds interfere with iron nutrition [8].

The phenylpropanoid pathway plays an important role in the biosynthesis of different groups of phenolic compounds [1]. The enzymes and related genes for branches of the pathway have been defined extensively in model plants [9–12]. Among the numerous phenotypic traits controlled by this pathway, pigmentation has been well characterized in several plant species. Generally, variability in black, purple, red, pink, brown, and yellow colouration in many tissues is the result of different combinations of the end products of this pathway [13]. A number of transcription factors (TFs) and modifying enzymes that influence gene expression in this pathway have been identified. The conserved TFs R2R3-MYB, WD-repeat (WDR), and basic-helix-loop-helix (bHLH) form an activation complex called the MYB-bHLH-WD (MBW) repeat complex [14] that controls the phenylpropanoid pathway in most plants.

Lentil (*Lens culinaris* Medikus) is an important grain legume crop that provides a good source of protein, carbohydrates, and micronutrients for humans. The primary seed coat colour in most market classes of lentil is determined by two independent genes: gray ground colour (*Ggc*) and tan ground colour (*Tgc*) [15]. The dominant and recessive allelic combinations of these two genes result in seed coats that are brown (*Ggc Tgc*), gray *(Ggc tgc)*, tan (*ggc Tgc*), or green (*ggc tgc*) and characterize specific market classes. Phenolic compound profiling of some lentil market classes has been reported [16–21], but there is no information about the specific phenolic compounds and associated phenylpropanoid pathway genes related to the basic set of lentil seed coat colours.

The lentil market class known as ‘zero-tannin’ is determined by expression of a single recessive gene, *tan* [22, 23]. Homozygous recessive *tan* is epistatic to *Tgc*, but not to *Ggc* [15]. In *tan* genotypes, the expression of the dominant *Ggc* produces a gray translucent seed coat, while the recessive *ggc* results in a transparent seed coat. The colour of seed coats in *tan* genotypes does not change during storage [24] or cooking. The thinner seed coat results in faster cooking, easier dehulling, and a rounder seed shape. These characteristics are desirable for processors and consumers, creating opportunities for breeding lentils with higher value. Zero-tannin seeds also imbibe water more quickly leading to imbibitional injury at the time of germination [24, 25], a negative agronomic characteristic that can be overcome using modified techniques such as seed coating.

The *tan* gene also influences pigmentation of stems and flowers. Non-mutant lentil plants have reddish stems, purple veins on floral tissues, and thicker, pigmented seed coats [22]. The *tan* phenotype is characterized by green stems and white flowers. This set of traits is similar to Mendel’s *A* gene in pea (*Pisum sativum*) [26], which encodes a bHLH TF that has a regulatory function with pleiotropic effects [27]. The absence of pigmentation in pea is the result of a mutation in this bHLH with mis-spliced mRNA caused by a premature stop codon [27]. The striking similarities between the two sets of phenotypes suggest that the lentil homologue of the pea *A* gene could be the lentil *tan* gene.

**Fig 1:**
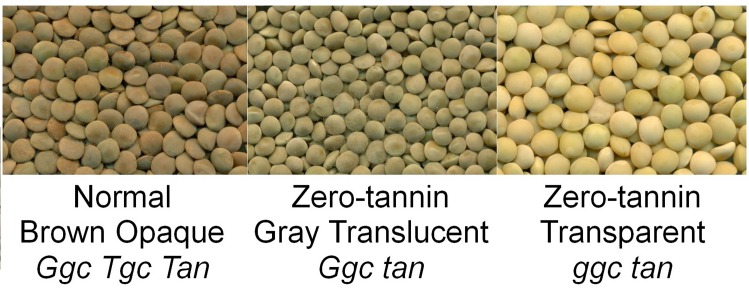
Images of lentil seeds with normal brown (*Ggc Tgc Tan*), zero-tannin gray (*Ggc tan*), and zero-tannin transparent (*ggc tan*) lentil seed coats.

The objective of this study was to compare the phenolic compound profiles obtained by liquid chromatography-mass spectrometry (LC-MS) of seed coats in the zero-tannin (*tan)* and normal *(Tan)* genotypes of lentil (Fig 1) along with the corresponding genotypic data. This information will help to further characterize *tan* as well as segments of the phenylpropanoid pathway that are influenced by this gene.

## Results

### Biochemical Analysis

The retention time and the optimized molecular and fragment ions of the standards and the potential compounds from different sub-classes of phenolic compounds are reported in Tables 1 and 2. Among the analyzed compounds, 4-hydroxybenzoic acid, chlorogenic acid, and *trans*-ferulic acid (phenolic acids sub-class), resveratrol aglycone (stilbenes sub-class), naringenin and flavanone aglycones (flavanones sub-class), apigenin, flavone, and luteolin-3',7-di-*O*-glucoside (flavones sub-class), kaempferol-3-*O*-glucoside, kaempferol-7-*O*-neohesperidoside, quercetin, quercetin-3-*O*-glucoside, quercetin-3-*O*-galactoside, quercetin-4'-*O*-glucoside, quercetin-3,4'-di-*O*-glucoside (flavonols sub-class), epigallocatechin gallate and epicatechin gallate (flavan-3-ols sub-class), procyanidin A_2_ and B_2_ (proanthocyanidin sub-class), and the anthocyanidins were not detected in any of the tested lentil samples.

**Table 1:** Phenolic compound characteristics in selected reaction monitoring (SRM), including sub-class, retention time, molecular ion, and optimum fragment ion in positive or negative mode.

Compound	Sub-class	Retention time (min)	Mode	Molecular ion (m/z)	Fragment ion (m/z)
Protocatechuic acid	Phenolic acids	3.8	Neg	153	109
Vanillic acid-4-*β*-*D*-glucoside	Phenolic acids	4.2	Neg	329	167
(−)-Gallocatechin	Flavan-3-ols	4.7	Neg	305	125
4-Hydroxybenzoic acid	Phenolic acids	5.0	Neg	137	93
Procyanidin B1	Proanthocyanidins	5.9	Neg	577	289
Catechin-3-glucoside*	Flavan-3-ols	6.5	Neg	451	137
(-)-Epigallocatechin	Flavan-3-ols	6.5	Neg	305	125
(+)-Catechin	Flavan-3-ols	6.6	Neg	289	203
±-Catechin-2,3,4-^13^C_3_	IS	6.6	Neg	292	206
Chlorogenic acid	Phenolic acids	6.9	Neg	353	191
Delphinidin-3-*β*-*D*-glucoside	Anthocyanidins	6.9	Neg	463	300
Procyanidin B2	Proanthocyanidins	7.1	Neg	577	289
(-)-Epicatechin	Flavan-3-ols	8.0	Neg	289	203
Dihydromyricetin	Dihydroflavonols	8.2	Neg	319	193
Kaempferol dirutinoside*	Flavonols	8.6	Neg	901	755
Cyanidin-3-O-rhamnoside	Anthocyanidins	8.7	Neg	431	285
*trans*-*p*-Coumaric acid	Phenolic acids	8.7	Neg	163	119
Malvidin-3-*O-*galactoside	Anthocyanidins	8.7	Neg	491	313
(-)-Epigallocatechin gallate	Flavan-3-ols	8.8	Neg	457	169
Quercetin-3,4'-di-*O*-glucoside	Flavonols	9.7	Neg	625	463
*trans*-Ferulic acid	Phenolic acids	9.9	Neg	193	134
Dihydroquercetin	Dihydroflavonols	10.2	Neg	303	125
Kaempferol-3-*O*-robinoside-7-*O*-rhamnoside	Flavonols	10.2	Neg	739	593
Luteolin-3',7-di-*O*-glucoside	Flavones	10.5	Neg	609	285
Procyanidin A2	Proanthocyanidins	10.5	Neg	575	285
Resveratrol-3-*β*-mono-*D*-glucoside	Stilbenes	10.5	Neg	389	227
(-)-Epicatechin gallate	Flavan-3-ols	10.6	Neg	441	169
Myricetin-3-O-rhamnoside	Flavonols	10.8	Neg	463	316
Quercetin-3-*O*-rutinoside	Flavonols	10.8	Neg	609	300
Quercetin-3-*O*-galactoside	Flavonols	11.0	Neg	463	300
Quercetin-3-*O*-glucoside	Flavonols	11.2	Neg	463	300
Luteolin-7-*O*-glucoside	Flavones	11.6	Neg	447	285
Kaempferol-3-*O*-rutinoside	Flavonols	11.8	Neg	593	285
Dihydrokaempferol	Dihydroflavonols	12.0	Neg	287	125
Kaempferol-3-*O*-glucoside	Flavonols	12.1	Neg	447	285
Quercetin-3-*O*-rhamnoside	Flavonols	12.2	Neg	447	300
Kaempferol-7-*O*-neohesperidoside	Flavonols	12.3	Neg	593	285
Apigenin-7-*O-*glucoside	Flavones	12.8	Neg	431	268
Quercetin-4′-*O*-glucoside	Flavonols	13.0	Neg	463	301
Myricetin	Flavonols	13.3	Neg	317	151
Luteolin-4'-*O*-glucoside	Flavones	13.4	Neg	447	285
Resveratrol	Stilbenes	13.6	Neg	227	143
Resveratrol-(4-hydroxyphenyl-^13^C_6_)	IS	13.6	Neg	233	149
4-Hydroxy-6-methylcoumarin	IS	14.0	Neg	175	131
Quercetin	Flavonols	15.5	Neg	301	151
Luteolin	Flavones	15.9	Neg	285	133
Naringenin	Flavanones	16.8	Neg	271	151
Kaempferol	Flavonols	17.6	Neg	285	187
Apigenin	Flavones	17.7	Neg	269	117
Flavone	Flavones	20.2	Pos	223	77
Flavanone	Flavanones	22.0	Pos	225	121

IS represents an internal standard.

Compounds without chemical standards are indicated with an *.

**Table 2:** Proanthocyanidin characteristics in single ion monitoring (SIM), including retention time and molecular ion in positive mode.

Composition	Sub-class	Retention time (min)	Mode	Molecular Ion (m/z)
GGC_I	Proanthocyanidins	4.7	Pos	899.7
GC_I	Proanthocyanidins	4.9	Pos	595.6
GGGC_I	Proanthocyanidins	5	Pos	1204.8
GGG	Proanthocyanidins	5.3	Pos	915.6
GGC_II	Proanthocyanidins	5.4	Pos	899.7
GCC_I	Proanthocyanidins	5.5	Pos	883.7
CC-gallate	Proanthocyanidins	5.5	Pos	731.7
GGGC_II	Proanthocyanidins	5.6	Pos	1204.8
GGCC_I	Proanthocyanidins	5.6	Pos	1187.8
GGC_III	Proanthocyanidins	5.7	Pos	899.7
GGGCC_I	Proanthocyanidins	5.7	Pos	1492.2
GC_II	Proanthocyanidins	5.9	Pos	595.6
GGCC_II	Proanthocyanidins	6.2	Pos	1187.8
GCCC_I	Proanthocyanidins	6.3	Pos	1171.8
GGGC_III	Proanthocyanidins	6.3	Pos	1204.8
GCC_II	Proanthocyanidins	6.4	Pos	883.7
GCC_III	Proanthocyanidins	6.5	Pos	883.7
±-Catechin-2,3,4-^13^C_3_	IS	6.6	Pos	294.2
GGGCC_II	Proanthocyanidins	6.9	Pos	1492.2
GCCCC_I	Proanthocyanidins	6.9	Pos	1460.1
CCCC_I	Proanthocyanidins	7.3	Pos	1155.9
GCCC_II	Proanthocyanidins	7.3	Pos	1171.8
CCCCC	Proanthocyanidins	7.9	Pos	1444.1
GCCCC_II	Proanthocyanidins	7.9	Pos	1460.1
CCCC_II	Proanthocyanidins	8.5	Pos	1155.9

IS, C, and G stand for internal standard, catechin/ epicatechin and gallocatechin/ epigallocatechin, respectively.

**Fig 2:**
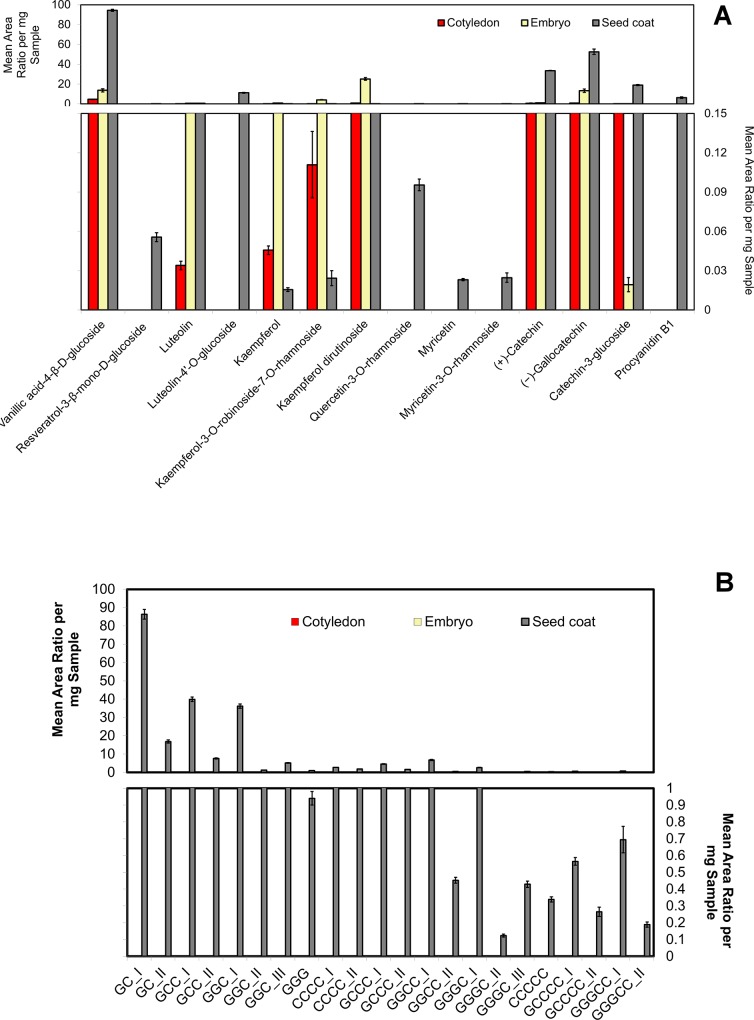
Mean area ratio per mg sample obtained for different sub-classes of phenolic compounds in (A) selected reaction monitoring (SRM) mode and (B) single ion monitoring (SIM) mode in cotyledon, embryo, and seed coat fractions of lentils with genotype *Ggc Tgc Tan;* C and G indicate catechin/ epicatechin and gallocatechin/ epigallocatechin, respectively.

In a preliminary experiment, analysis of variance showed no significant differences between the RILs within either the same phenotypic groups of normal brown opaque seed coats (genotype *Ggc Tgc Tan*) or gray translucent zero-tannin seed coats (genotype *Ggc tan*) for most of the analyzed phenolic compounds (S1 and S2 Tables). In a second preliminary experiment, the phenolic profiles of the three seed fractions (cotyledon, seed coat, and embryo) of the gray seed coat of CDC Maxim were significantly different (Fig 2A and 2B). Vanillic acid-4-*ß*-*D*-glucoside, luteolin, kaempferol glycones and aglycone, and flavan-3-ols (including catechin, gallocatechin, and catechin-3-glucoside) were detected in all three seed fractions, but resveratrol-3-*ß*-mono-*D*-glucoside, luteolin-4'-*O*-glucoside, quercetins, myricetins, and oligomers of proanthocyanidins (i.e., dimers, trimers, tetramers, and pentamers) were detected only in the seed coat fraction.

**Fig 3:**
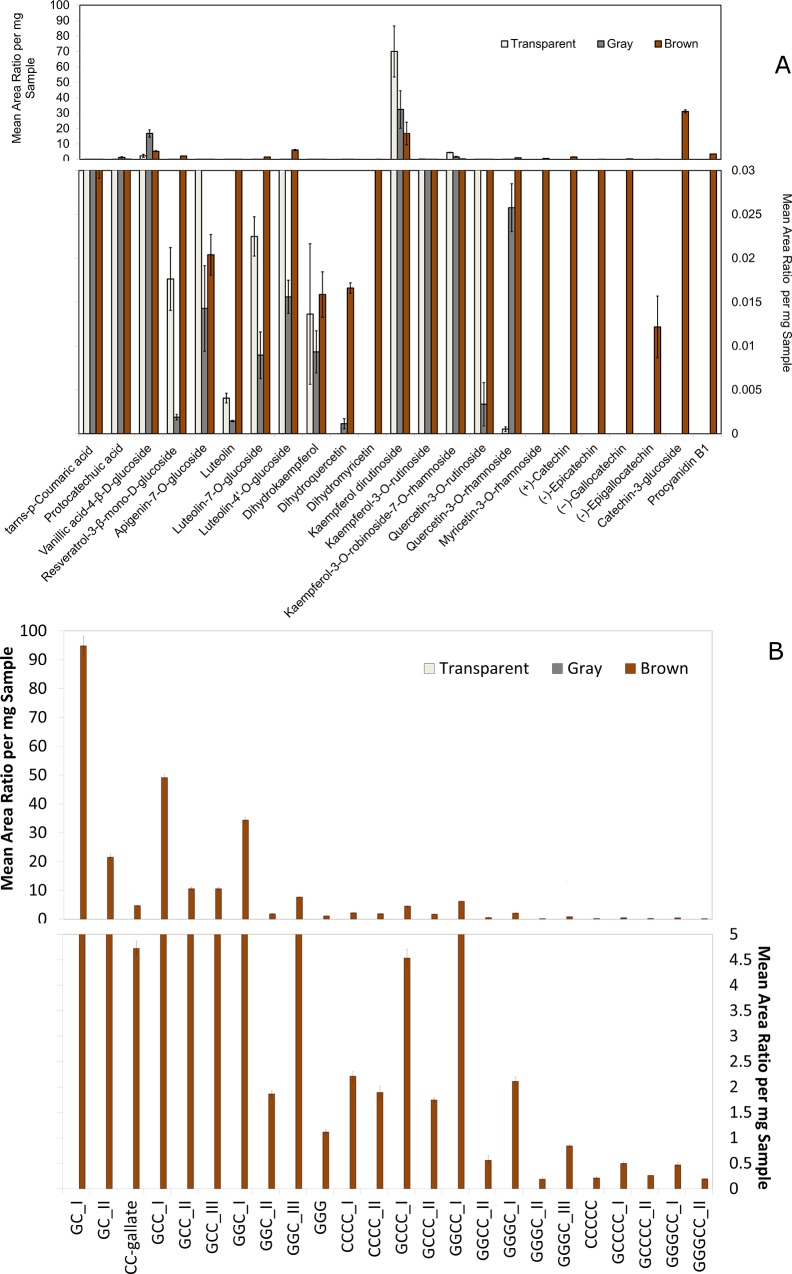
Mean area ratio per mg sample obtained for different sub-classes of phenolic compounds in (A) SRM mode and (B) SIM mode for transparent, gray translucent, and brown opaque lentil seed coats; C and G stand for catechin/ epicatechin and gallocatechin/ epigallocatechin, respectively.

Based on the results of the two preliminary tests, further investigations into the phenolic profile for seed coats that were normal brown opaque (*Ggc Tgc Tan*), gray translucent zero-tannin (*Ggc tan*), and transparent zero-tannin (*ggc tan*) were conducted. Among the phenolic acids, *trans-p*-coumaric acid, protocatechuic acid, and vanillic acid-4-*β-D*-glucoside were detected in all three phenotypes (Fig 3A). Resveratrol-3-*β*-mono-*D*-glucoside and flavones, including apigenin-7-*O*-glucoside and luteolin aglycone and glycones, were found in all three seed coat types. Among the dihydroflavonols, dihydrokaempferol was found in all three seed coat phenotypes while dihydroquercetin was predominantly found in the brown opaque and to a lesser amount in the gray translucent seed coats. Dihydromyricetin was detected only in the gray zero-tannin phenotype. Kaempferol glycones were detected in all three seed coat phenotypes. Quercetin-3-*O*-rutinoside was detected at a low level in gray phenotypes, while quercetin-3-*O*-rhamnoside was at a very low level in transparent zero-tannin. Myricetin-3-*O*-rhamnoside, however, was found only in the brown opaque phenotype. Flavan-3-ols including catechin, epicatechin, gallocatechin, epigallocatechin, and catechin-3-glucoside were observed only in brown opaque seed coats. Similar results were observed for proanthocyanidin dimers, trimers, tetramers, and pentamers (Fig 3A and 3B).

### Molecular Analysis

**Fig 4:**
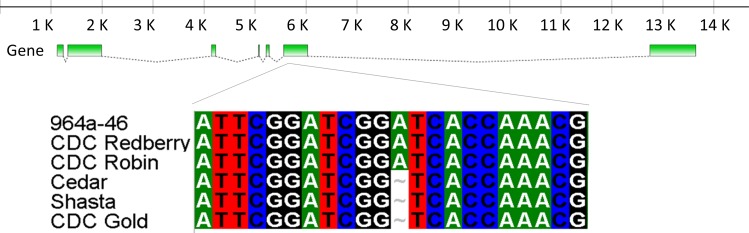
Structure of *LcubHLH* highlighting the region of the variant related to *tan*. The *tan* lines (Cedar, Shasta and CDC Gold) all have a deletion relative to the *Tan* lines (964a-46, CDC Redberry, and CDC Robin). The gene structure and the sequence alignment were obtained by FancyGene [28] tool and BioEdit [29] alignment software, respectively.

The SNP marker LcC01900p336 was found in a contig that was homologous to the 3’ end of the *A* gene of pea. It was polymorphic between the parents of LR-30 and it co-segregated with the seed coat phenotype in the segregating RILs (S3 Table). When tested on a panel of 96 lines, however, the genotyping results did not correlate with the phenotypes (data not shown), suggesting it is not the causative mutation and is simply genetically linked in the LR-30 population. Sequencing through the exonic regions of this gene in multiple *tan* and *Tan* lines revealed a common SNP in all three *tan* lines that was not found in the *Tan* lines (Fig 4). The gene consisted of seven exons and this SNP, at position 343 in exon 6, introduces a premature STOP codon that would result in a truncated protein and a non-functioning enzyme. It should be noted that the mutation in pea that causes the white flower character is caused by an SNP in the splice site at the end of Exon 6, ~165 bp after this deletion. The KASP assay LcZT-Exon6p343, designed to test for this SNP, consistently identified all *tan* lines and demonstrated that none of the *Tan* lines have the variant allele in the mapping population LR-30 (S3 Table and S1 Fig) and in the diversity panel (S4 Table and S2 Fig).

## Discussion

Phenolic compounds are produced through the actions of numerous regulatory genes and TFs in the phenylpropanoid pathway. These compounds fulfill different roles including seed pigmentation for plants and confer health benefits to humans who eat the seeds [2–6]. We used a combination of biochemical and genetic approaches to investigate the phenylpropanoid pathway to elucidate what is responsible for the lack of seed coat pigmentation in zero-tannin (*tan*) lentil phenotypes. To accomplish this, we compared seed coats from brown opaque (*Ggc Tgc Tan*), gray translucent zero-tannin (*Ggc tan*), and transparent zero-tannin (*ggc tan*) phenotypes.

**Fig 5:**
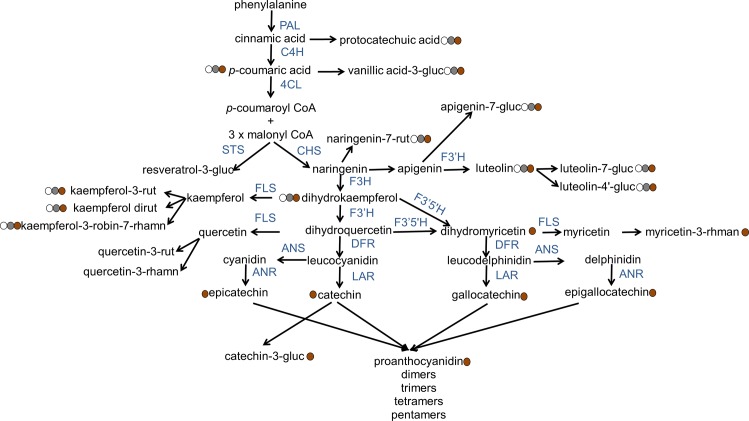
Putative biochemical pathway of phenolic compounds in lentil seed coats, as well as whether or not the final product of different branches of phenylpropanoid are observed in *Tan* and/ or *tan* genotypes. Abbreviations: PAL, phenylalanine ammonia lyase; C4H, cinnamic acid 4-hydroxylase; 4CL, 4-coumaric acid:CoA ligase; STS, stilbene synthase; CHS, chalcone synthase; F3H, flavanone-3-hydroxylase; F3’H, flavonoid-3’-hydroxylase; F3’5’H, flavonoid-3’,5’-hydroxylase; FLS, flavonol synthase; DFR, dihydroflavonol reductase; ANS, anthocyanidin synthase; LAR, leucoanthocyanidin reductase; ANR, anthocyanidin reductase; gluc, glucoside; rut, rutinoside; robin, robinoside; rhamn, rhamnoside. Filled circles represent the transparent, gray, and brown seed coat colours. Information with respect to the pathway originates from [9–12].

The most obvious difference between the *Tan* and *tan* genotypes was the presence of dihydromyricetin, myricetin-3-*O-*rhmanoside, flavan-3-ols, and proanthocyanidin oligomers in the brown lines and the absence of these in the zero-tannin phenotypes (Fig 3A and 3B). We layered our phenolic compound profile results on the pathway suggested in previous literature [9–12] and present them as a putative biochemical pathway in Fig 5. Dihydromyricetin can be produced by F3’5’H from dihydroquercetin and/ or dihydrokaempferol (Fig 5). Myricetin, gallocatechin/ epigallocatechin (from flavan-3-ols), and several proanthocyanidins should be produced from dihydromyricetin in subsequent steps; however, none of these phenolic compounds were detected in the zero-tannin phenotypes. This shows that the phenylpropanoid pathway in these phenotypes is being blocked at the point where *F3’5’H* acts. Furthermore, catechin/ epicatechin requires dihydroquercetin as a precursor, and therefore the phenylpropanoid pathway should also be blocked at the location of *DFR* activity. In *Brassica carinata* seeds, dihydrokaempferol, dihydroquercetin, and trace amounts of dihydromyricetin accumulate in yellow-seeded (i.e., transparent seed coat) phenotypes, while proanthocyanidins are observed only in brown-seeded phenotypes [30]. The level of mRNA for *flavanone-3-hydroxylase* (*F3H*) and *flavonoid-3’-hydroxylase* (*F3’H*) is similar between dark and transparent seed coats of *B*. *rapa*. However, the amounts of mRNA for *DFR*, *anthocyanidin synthase* (*ANS*), and *anthocyanidin reductase* (*ANR*) are not statistically significant in transparent seed coat phenotypes [31]. *Arabidopsis thaliana tt3* mutant seeds have transparent seed coats, and visible anthocyanidins or proanthocyanidins are not detected in the *tt3* mutant because it lacks *DFR* mRNA [32]. Strong down-regulation of genes such as *ANR* and *ANS* lead to reduced amounts of proanthocyanidins and anthocyanidins and a transparent seed coat in *Medicago truncatula* [33].

Our molecular analyses confirm that *tan* is most likely a bHLH, orthologous to the *A*-gene in pea. The LcZT-Exon6p343 allele found in *tan* lentil genotypes introduces a premature STOP codon that prevents the expression of a full copy of bHLH. As *tan* is epistatic to *Tgc*, the tan ground colour is not observed in *Ggc Tgc tan* or *ggc Tgc tan* genotypes. The gene *MtTT8* in *M*. *truncatula* (which is a homologous bHLH) controls pranthocyanidin- and anthocyanidin-related genes such as *ANR* and *ANS* [33]. A large insertion mutation in *BrTT8* results in transparent phenotype in *B*. *rapa*. This bHLH controls the expression of *ANS* and *ANR* [31]. The allele *tt3* (*DFR*) in *A*. *thaliana* seeds with transparent seed coats is controlled by a group of TFs including bHLHs such as TT8 [34]. TT2 (R2R3-MYB protein) and TTG1 (WDR protein) control *DFR*, showing that they also interact to control the phenylpropanoid pathway genes. A ternary MBW complex has been proposed for controlling the late sections of the phenylpropanoid pathway [34]. Therefore *tan*, as a bHLH part of this MBW complex, interacts with the regulatory genes in the phenylpropanoid pathway starting from *F3’5’H* and *DFR*.

All of the analyzed phenolic acids are found in all three lentil seed coat colours (Fig 3A). Among the flavonols, kaempferols are found in all three seed coat phenotypes tested. The aglycone and glycones of kaempferols are also observed in all three seed fractions (i.e., embryo, cotyledon, seed coat) (Fig 2A). However, the remaining flavonols, including quercetins and myricetins, are detected only in the seed coat (Fig 2A). Our analysis did not detect dihydroquercetin in the transparent phenotype; however, quercetin glycones are found in all three lentil seed coat colours. This suggests that dihydroquercetin should be present in the transparent seed coat. Because the signal intensity of the dihydroquercetin in the brown seed coat was observable but weak, this compound appears to be in low abundance and likely below the low detection limit in the transparent lentil phenotype.

The phenylpropanoid pathway affects plant characteristics other than pigmentation, including protection of the plant against stresses. Although seed coat phenolics can provide a good barrier against pathogens, the embryo and cotyledons need to be protected by chemical defense materials such as phenolic compounds when the seed starts germinating and the barrier ruptures [35]. Dueñas et al. (2002) reported that catechin and various phenolic acids were present in lentil cotyledons [18]. However, they did not report a variety of flavonoids in cotyledons of lentils. Our study detected a diversity of phenolic compounds, including phenolic acids, flavones, flavonols, and different flavan-3-ols, in cotyledon and embryo fractions. All or some of these compounds may play a role in the protection of the embryo and cotyledons.

Zero-tannin lentils do not change colour during storage [24], likely due to the lack of flavan-3-ols and proanthocyanidins. We previously reported a significant reduction in flavan-3-ols and proanthocyanidins due to polymerization of these compounds in lentils that were stored in the dark for long periods of time [36]. However, phenolic compounds improve seed establishment [37], and as a result their reduction might introduce problems with damage caused by rapid water imbibition during germination [24, 25]. Furthermore, zero-tannin lentils are more susceptible to soil- and seed-borne diseases, a problem that must be circumvented by using seed-applied fungicides [38].

Flavan-3-ols such as catechin and gallocatechin show anti-inflammatory and anti-oxidative activity and have been associated with the reduction of some cardiovascular diseases [39, 40]. Proanthocyanidins are the major antioxidants entering the colon [5], and they may reduce cholesterol [40], inhibit the growth of breast cancer cells [41], and protect the prostate [42]. Zero-tannin lentils cannot provide the health benefits associated with flavan-3-ols and proanthocyanidins; but some health advantages can be achieved. Phenolic acids, flavones, and flavonols show anti-oxidative [2–4], anti-cardiovascular disease [3], anti-cancer [3–6], anti-diabetic [3, 6], and anti-HIV [3, 6] effects. They may also increase the bioavailability of iron [8]. Our new knowledge of the underlying basis of the genotypes and phenotypes of zero-tannin lentil seed coats will be useful for designing future lentil cultivars with improved nutritional profiles.

## Materials and Methods

### Plant Material

Lentil recombinant inbred line (RIL) population LR-30, which consists of 138 lines, was derived from a cross between the brown seed coat cultivar CDC Robin (genotype *Ggc Tgc Tan*) and a zero-tannin plant from the breeding line 2670b (genotype *Ggc Tgc tan*). Both genotypes are homozygous for *Tgc* and the RILs of this population have either normal brown or gray zero-tannin seed coats based on segregation of the dominant or recessive alleles at the *Tan* locus. Seed coats of RILs were phenotyped visually and classified as brown opaque (*Ggc Tgc Tan*) or zero-tannin gray translucent (*Ggc Tgc tan*).

In a preliminary test, two subsets of 10 RILs of each phenotype were randomly selected for biochemical analysis of the phenolics profile of the lentil seeds. Whole seeds of these 20 RILs were obtained from three biological replicates grown in a randomized complete block design in the field in 2013 at Saskatoon, SK, Canada.

In a second preliminary test, one available gray seed coat normal genotype, CDC Maxim (*Ggc tgc Tan*), was decorticated and seed coats were separated from cotyledons and embryos [43]. All three seed fractions were similarly analyzed with three technical replicates.

Based on the preliminary analyses, one representative RIL from the *Ggc Tgc Tan* genotype group (LR-30-76) and one representative from the *Ggc Tgc tan* genotype group (LR-30-98) were compared with seed coats of a *ggc tan* genotype (CDC Gold) (Fig 1). CDC Gold has a transparent seed coat that allows its cotyledon colour to be easily observed. Seeds of CDC Gold were also produced in the field in 2013 at Saskatoon.

The seeds of all three genotypes were decorticated to obtain the seed coat fractions that were analyzed using three technical replicates.

### Reagents and Standards

Tables 2 and 3 show a complete list of the phenolic compounds analyzed in this study, including sub-classes of phenolic acids, stilbenes, anthocyanidins, flavan-3-ols, proanthocyanidins, flavanones, flavones, dihydroflavonols and flavonols. The majority of the phenolic compounds investigated in this experiment were identified previously (S1 Text) and [43]. Additional standards investigated in this work included protocatechuic acid, 4-hydroxybenzoic acid, chlorogenic acid, *trans-p-*coumaric acid, *trans*-ferulic acid, dihydroquercetin, dihydrokaempferol, dihydromyricetin, resveratrol-(4-hydroxyphenyl-^13^C_6_), cyanidin-3-*O*-rhamnoside, and resveratrol. All standards were purchased from Sigma-Aldrich (Missouri, USA) except cyanidin-3-*O*-rhamnoside, which was purchased from Extrasynthese (Genay, France). The two compounds labelled with an asterisk (*) in Table 1 (catechin-3-glucoside and kaempferol dirutinoside) are not commercially available, but have been previously identified in lentil seed [17, 19] and putatively identified in our previous work (S1 Text) and [43]. The proanthocyanidins in Table 2 did not have commercially available standards, and therefore the order of C’s (catechin or epicatechin) and G’s (gallocatechin or epigallocatechin) is arbitrarily assigned for these oligomers.

**Table 3:** Mobile phase gradient used in this experiment, where solvent A and B were water: formaic acid (99;1, v/v) and water: acetonitrile: formic acid (9:90:1, v/v/v), respectively.

Time (min)	A%	B%	Flow (mL/min)
0	99	1	0.35
1	99	1	0.35
21	59	41	0.35
24	40	60	0.35
24.1	20	80	0.35
26	20	80	0.35
26.1	99	1	0.35
30	99	1	0.35

### Sample Preparation

For each replicate, 1000 μL of the extraction solvent (acetone:water, 70:30 v/v) containing the internal standards was added to 250 mg of freeze-dried sample in micro-centrifuge tubes. The internal standards are added to account for changes in the matrix among the cultivars and enables relative quantification [44] to be used when comparing the phenolic profiles among the cultivars. When separate seed fractions (cotyledons, embryos, and seed coats) were analyzed, the extraction solvent and the freeze-dried samples were reduced to 250 μL and 50 mg, respectively. Samples were crushed into a fine paste using a Fast Prep®FP120 (Qbiogene, Inc., Canada) with a maximum of seven consecutive times for 45 s each at a speed setting of 4.0. Samples were shaken for 1 h on a rocking platform at a speed of 1400 rpm. The tubes were centrifuged twice (12,000 rpm for 5 min each) and 100 μL of the supernatant was dried down with a Speed Vac (LABCONCO, Kansas City, USA). Dried samples were then reconstituted in 100 μL methanol: water (10:90, v/v) (S1 Text).

### HPLC-MS

Previously optimized chromatographic conditions [43] were applied with some modifications. The LC hardware was an Agilent 1290 UPLC equipped with a G4226A autosampler, a G4220 A binary pump, a G1316 TCC, and a G4212 DAD detector. The column was a Core-shell Kinetex pentafluorophenyl (PFP) (100 mm × 2.1 mm id), with 2.6 μm particle size (Phenomenex, Torrance, CA). The mobile phases were water:formic acid (FA) (99:1, v/v) as solvent A and water:acetonitrile (ACN):FA (9:90:1, v/v/v) as solvent B. The same solvent gradient was employed as previously reported (Table 3) [43]. Although some retention times were slightly earlier than in our previous study, this was readily attributed to the smaller mixing volume of the Agilent 1290 UPLC compared with the Agilent 1100 HPLC. Relative quantification was determined for phenolic compounds (Table 1) using selected reaction monitoring (SRM) and for proanthocyanidins (Table 2) using single ion monitoring (SIM). Peak areas of each analyte were integrated with Thermo Xcalibur 2.1 software and normalized to the peak area of a related internal standard (IS). Values are reported per mg of dry sample.

### Statistical Analysis

Analysis of variance and means comparisons of area ratio per mg of sample were done using R software (v. 2.15.3) [45]. Duncan’s multiple range test was used for comparing the means of area ratio per mg samples (95% confidence level).

### Molecular Markers

To initially test if the lentil homologue of the *A*-gene of pea segregated with *tan*, the nucleotide sequence for the pea gene [GU132941] was used to tBLASTx an in-house collection of 3’ transcript sequences of lentil from which SNPs had been identified [46]. A number of sequences from various lentil lines matched the pea sequence. An alignment of these sequences, using BioEdit [29] alignment software, revealed an SNP (LcC01900p336) located 52 nucleotides downstream from the STOP codon. A KASP assay (LGC Genomics, Hoddesdon, UK) was designed to assay genotypes at this SNP. The allele specific primers were A1 = GAAGGTGACCAAGTTCATGCTGACAAAATCACGTGATGTTGTGACTC and A2 = GAAGGTCGGAGTCAACGGATTGACAAAATCACGTGATGTTGTGACTT. The conserved primer was C1 = AAGCCAATGTGTACCAATGATGTATCATTA. DNA was extracted from a single individual of each LR-30 RIL using a modified CTAB extraction [47]. Assay reaction volume was 10 μL of 50 ng/μL DNA, 2X KASP Reaction Mix, and 0.17 μM KASP Assay Mix (allele-specific primers, A1 and A2, and common primer, C1). PCR amplification was carried out in a StepOnePlus^Tm^ Real-Time PCR System (Applied Biosystems, California, US) and fluorescence was analyzed using StepOne Software version 2.1 (Applied Biosystems, California, US).

To identify the putative causal mutation in the gene, the pea sequence was compared to a preliminary assembly of the lentil genome (CDC Redberry v0.3) using tBLASTx to identify the full lentil homologue. Nested primers were designed to span the introns across the full gene and to amplify fragments from several *Tan* and *tan* lines. Amplified fragments were run out on a 1% agarose gel, bands were cut out and purified using a Qiagen gel extraction kit (cat.no. 28706), and the resulting DNA sequenced using the Sanger method. Sequences were aligned to the reference genome (CDC Redberry v0.3) and SNPs identified using BioEdit alignment software.

A KASP assay, LcZT-Exon6p343, with

A1 = GAAGGTGACCAAGTTCATGCTGCCCGATGATATTCGGATCGGA,

A2 = GAAGGTCGGAGTCAACGGATTGCCCGATGATATTCGGATCGGT, and

C = GGCCAACAAATGAAAATCTGAGTCCAAAT), was designed for a candidate SNP and used to survey a panel of 96 lentil genotypes (S4 Table) representative of a wide range of seed coat colours and patterns and the zero-tannin cultivars Cedar, Shasta, CDC Zt-4, and CDC Gold.

## Supporting Information

S1 Fig KASP assay results for LcZT-Exon6p343 on LR-30 RILS and parents. (XLSX)

S2 Fig KASP assay results for LcZT-Exon6p343 on a panel of diverse lentil lines. (XLSX)

S1 Table Analysis of variance for 10 brown normal *Ggc Tgc Tan* lines. ** and *** denote significance at the 0.01 and 0.001 probability levels, respectively.
(XLSX)

S2 Table Analysis of variance for 10 gray zero-tannin *Ggc Tgc tan* lines in three replicates. (XLSX)

S3 Table Segregation matrix for SNP loci and phenotypes on LR-30 RILs and parents. (XLSX)

S4 Table The panel of diverse lentil lines. (XLSX)

S1 Text Mirali et al., Unpublished data. (DOCX)
