# Supplementary material for: Genetics and Biochemistry of Zero-Tannin Lentils
Source: PLoS One. 2016 Oct 27;11(10):e0164624. doi: 10.1371/journal.pone.0164624 (PMC5082924; doi:10.1371/journal.pone.0164624)
Supplement: S1 Text — (DOCX) [file pone.0164624.s007.docx]

**Profiling the Phenolic Compounds of the Four Major Seed Coat Types in Lentil**

Mahla Mirali *, Randy W. Purves, Albert Vandenberg

Plant Sciences Department, University of Saskatchewan, Saskatoon, SK, Canada S7N 5A8

Corresponding author’s E-mail: [mam194@mail.usask.ca](mailto:mam194@mail.usask.ca) and [mahlamirali@gmail.com](mailto:mahlamirali@gmail.com)

ABSTRACT

Phenolic compounds can provide antioxidant health benefits for humans, and foods such as lentil can be valuable dietary sources of different sub-classes of these secondary metabolites. This study used liquid chromatography-mass spectrometry (LC-MS) analyses to compare the phenolic profiles of lentil genotypes with four seed coat background colours (green, gray, tan, and brown) and two cotyledon colours (red and yellow) grown at two locations. The mean area ratio per mg sample (MARS) values of various phenolic compounds in lentil seeds varied with the different seed coat colours conferred by specific genotypes. Seed coats of lentil genotypes with the homozygous recessive *tgc* allele (green and gray seed coats) had higher MARS of flavan-3-ols, proanthocyanidins, and some flavonols. This suggests lentils featuring green and gray seed coats might be more promising as health-promoting foods.

Keywords

Lentil; Seed coat colour; LC-MS; Polyphenol; Flavan-3-ol; Proanthocyanidin; Flavonol

Abbreviations

RIL, recombinant inbred line; STH, Sutherland; SPG, Saskatchewan Pulse Growers; ESI, electrospray ionization; IS, internal standard; PFP, pentafluorophenyl; FA, formic acid; ACN, acetonitrile; MRM, multiple reaction monitoring; SIR, single ion recording; C, catechin/epicatechin; G, gallocatechin/epigallocatechin; MARS, mean area ratio per mg sample

1. Introduction

Phenolic compounds are a large group of secondary metabolites characterized by an aromatic ring connected to OH groups. They are produced via the metabolic pathways of shikimate, phenylpropanoid, and acetate. Simple phenol, phenolic acids, stilbenes, and flavonoids (flavanones, flavan-3-ols, flavones, flavonols, anthocyanidins, isoflavones, and tannins, including condensed tannins or proanthocyanidins) are the major sub-classes of phenolic compounds.(Vermeris and Nicholson, 2006) These compounds play important roles in plants, including pigmentation and defense against pathogens and abiotic stresses.(Andersen and Jordheim, 2010) As components of the human diet, the benefits of polyphenols (e.g., anti-oxidant properties) have been an area of interest.(Andersen and Jordheim, 2010) Lentil *(Lens culinaris* Medikus) is an ancient crop that originated in the Near East but now is consumed worldwide. Lentil is a good source of protein, carbohydrates, minerals, vitamins, and secondary metabolites such as phenolics.(Xu and Chang, 2009) Various phenolic compounds including phenolic acids, stilbenes, and several types of flavonoids have been detected in lentil.(Aguilera et al., 2010; Bartolomé et al., 1997; Dueñas et al., 2002; Dueñas et al., 2003; López-Amorós et al., 2006)

Lentil seed coats have a wide range of background colour and patterns. The seed coat ground colour in lentil is mainly determined by two independent genes: *Ggc* and *Tgc*(Vandenberg and Slinkard, 1990)*.* The dominant and recessive combinations of two alleles at each locus determines the four basic seed coat ground colours known as brown (*Ggc Tgc*), gray *(Ggc tgc)*, tan (*ggc Tgc*), and green (*ggc tgc*)(Vandenberg and Slinkard, 1990). Furthermore, a single gene controls the inheritance of red vs. yellow cotyledon colour in lentil; the genetic combination *Yc* will produce a red cotyledon while *yc yc* produces a yellow cotyledon.(Slinkard, 1978)

Information about how the background colour genes in lentil are related to specific phenolic compound profiles has not been reported. Therefore, the objective of this study was to compare the phenolic compounds profile acquired by liquid chromatography-mass spectrometry (LC-MS) for green, gray, tan, and brown seed coat ground colour phenotypes of lentil with either red or yellow cotyledons and determine if they are characteristic of specific genetic combinations of the alleles of the *Ggc* and *Tgc* loci.

2. Materials and methods

**2.1. Plant Material.** Seeds of lentil recombinant inbred line (RIL) population LR-18 (Tar'an et al., 2003) were obtained from fresh seed lots grown in 2013 at the Crop Development Centre (University of Saskatchewan) at the Sutherland (STH) and Saskatchewan Pulse Growers (SPG) farms near Saskatoon, Canada. The parents of LR-18 are CDC Robin (brown seed coat with red cotyledon) and 964a-46 (pale green seed coat with yellow cotyledon). The population segregates independently for *Ggc, Tgc*, and *Yc* alleles producing brown, gray, tan, and green seed coat colours and also red and yellow cotyledon colours. For each seed coat colour combination, seed samples of a subset of eight RILs (four yellow and four red cotyledons) from the LR-18 population were randomly selected (Table 1). Seeds of three replications of each genotype grown at both locations were analyzed. Seeds were homogeneous for plumpness and diameter and had no evidence of seed coat pattern. Furthermore, the football fractions (entire decorticated seeds) of two RILs with green seed coat/yellow cotyledon and two RILs with green seed coat/red cotyledon (obtained in three biological replicates) were compared.

**2.2. Reagents and Standards**. Tables 2 and 3 show the phenolic compounds analyzed, including sub-classes of phenolic acids, stilbenes, anthocyanidins, flavan-3-ols, proanthocyanidins, flavanones, flavones, and flavonols. The analysis method was previously optimized(Mirali et al., 2014), but new compounds were added, including flavanone, myricetin, kaempferol, kaempferol-3-*O*-robinoside-7-*O*-rhamnoside, kaempferol-3-*O*-rutinoside, quercetin-4'-*O*-glucoside, vanillic acid-4-*ß*-*D*-glucoside, apigenin-7-*O*-glucoside, and (±)-catechin-2,3,4-^13^C_3_, which were purchased from Sigma-Aldrich (Missouri, USA). Resveratrol-3-*ß*-mono-*D*-glucoside and delphinidin-3-*ß*-*D*-glucoside were obtained from Santa Cruz Biotechnology, Inc. (California, USA) and (-)-epicatechin gallate, (-)-epigallocatechin, (-)-epigallocatechin gallate, luteolin-3',7-di-*O*-glucoside, kaempferol-7-*O*-neohesperidoside, quercetin, quercetin-3-*O*-rhamnoside, quercetin-3-*O*-rutinoside, quercetin-3,4'-di-*O*-glucoside, apigenin, (-)-epicatechin, cyanidin-3,5-di-*O*-glucoside, procyanidin B2 (epicatechin-(4*β*→8)-epicatechin), and procyanidin A2 (epicatechin-(2*β*→7,4*β*→8)-epicatechin) were purchased from Extrasynthese (Genay, France). Kaempferol- and catechin-glycones (Table 2) and several oligomers of proanthocyanidins (Table 3) found in the lentil seed matrix, but that did not exist commercially, were analyzed based on previous reports.(Aguilera et al., 2010; Dueñas et al., 2003; Mirali et al., 2014) In Table 3, C and G stand for catechin or epicatechin and gallocatechin or epigallocatechin, respectively. Note that the order of Gs and Cs given is arbitrary.

**2.3. Sample Preparation.** Samples were prepared based on previous sets of optimization tests(Mirali et al., 2014) with minor modifications. In summary, for each replicate 1000 µL of the extraction solvent (acetone:water (70:30 v/v)) was added to ~250 mg freeze-dried lentil sample in a micro centrifuge tube. By adding two ¼ inch ceramic sphere beads to each tube, samples were crushed to a fine paste using a Fast Prep®FP120 (Qbiogene, Inc., Canada) for a maximum of seven consecutive times of 45 s each at a speed setting of 4.0. Samples were shaken for 1 h on a rocking platform at a speed of 1400 rpm. The tubes were centrifuged twice (12,000 rpm for 5 min each) and 100 µL of the supernatant was dried down with a Speed Vac (LABCONCO, Kansas City, USA). Dried samples were then re-dissolved and reconstituted in 100 µL methanol: water (10:90, v/v).

**2.4. HPLC-MS.** Previously optimized chromatographic condition (Mirali et al., 2014) was applied using an Alliance 2695 (Waters, UK) reversed phase HPLC with 996 PDA UV/VIS detector coupled to a Quattro Ultima (Waters, UK) triple quadrupole MS equipped with an electrospray ionization (ESI) interface. The peak area of each analyte was integrated with Waters’ MassLynx 4.1 software and normalized to the peak area of a related internal standard (IS). The chromatographic column was a Core-shell Kinetex pentafluorophenyl (PFP), 100 × 2.1 mm id, 2.6 μm particle size (Phenomenex, Torrance, CA). H_2_O:formic acid (FA) (99:1, v/v) as solvent A and H_2_O:acetonitrile (ACN):FA (9:90:1, v/v/v) as solvent B were used for mobile phases at a flow rate of 0.35 mL/min and a gradient detailed in Table 4. The column oven temperature was 40 °C and the injection volume was 2 µL. Quantification of phenolic compounds was done using multiple reaction monitoring (MRM) and single ion recording (SIR) in positive mode. For the MRM, eight functions were defined with time ranges of 0-7, 2.5-12, 4-20, 6-15, 9.5-17, 11.5-20, 15.5-25, and 20-29.9 min in the mass spectrometry software; for the SIR, one function with several transitions was used. The reproducibility of the LC-MS method was confirmed as described previously.(Mirali et al., 2014)

**2.5. Statistical Analysis.** Data were analyzed using a linear mixed model using the *lmerTest* package (Kuznetsova et al., 2016) in R (v. 3.2.4).(R Core Team, 2016) The best model was fit considering location as random effect and seed coat colour, cotyledon colour, and their interaction as fixed effects. Appendix Tables S1-S3 show the *P*-values from mixed model ANOVA F-test for the response variables. Location showed significant effect on resveratrol-3-*ß*-mono-*D*-glucoside, catechin, gallocatechin, procyanidin B1, GGC_I, GGC_II, GGG, GGCCs, GGGCs, GCCCC_I and GGGCCs. Therefore, these compounds were analyzed separately for SPG (Appendix Table S1) and STH (Appendix Table S1) locations. For the rest of phenolic compounds, data from two locations were combined (Appendix Table S3).

3. Results

Table 2 shows the optimization results for retention time (Rt), molecular and fragment ions, and UV wavelength of the standards and potential candidates from different sub-classes of phenolic compounds. An example of the overlaid chromatograms of the detected peaks in a gray seed coat/red cotyledon lentil genotype (RIL group # 3) is provided in Figure 1; chromatograms for other seed coat/cotyledon combinations were similar. MS analysis was used to quantify the expected phenolic compounds, while UV was used to identify unexpected phenolics in the range from 250 to 600 nm(Mirali et al., 2014).

Vanillic acid-4-*ß*-*D*-glucoside (peak 1) from the phenolic acids sub-class eluted at an Rt of 2.8 min (Table 2; Figure 1) and was observed for all four lentil seed coat colours. The optimum molecular ion was protonated [M+H]^+^ at m/z 331, and the fragment ion at m/z 169 was related to loss of glucose. The UV spectra for this compound featured two major peaks at 254 and 291 nm.

Resveratrol-3-*ß*-mono-*D*-glucoside (peak 10) from the stilbenes sub-class was also detected in all lentil background colours (Table 2; Figure 1). The optimum molecular ion was protonated [M+H]^+^ at m/z 391, and loss of glucose caused a fragment ion at m/z 229. The maximum peak in the UV spectrum was at 321 nm.

*Flavanones*. Neither naringenin nor flavanone were detected in any of the brown, gray, tan, or green lentil samples (Table 2).

*Flavones*. Several members of the flavones sub-class were investigated (Table 2), and the UV spectra showed two major peaks at 254-268 and 298-358 nm, respectively, for this sub-class. Peak 14 corresponded to luteolin-4'-*O*-glucoside(Mirali et al., 2014), which was detected in all four seed coat types. The remainder of the flavones were not detected in our lentil samples.

*Flavonols*. Aglycones and glycones of quercetin, myricetin, and kaempferol were surveyed in all lentil samples (Table 2). Peak 8 corresponds to the UV spectra of kaempferol (266, 346 nm) (Table 2; Figure 1). It had a molecular ion [M+H]^+^ at m/z 904 and its fragment ion at m/z 433 ([M+H-162-162-146]) indicated a loss of two hexoses and one rhamnose. The compound was identified as kaempferol dirutinoside.(Aguilera et al., 2010) Peak 11 eluted at 11.9 min and the molecular ion to fragment ion transition from 742 to 433 transition identified this compound as kaempferol-3-*O*-robinoside-7-*O*-rhamnoside. Peak 12 corresponded to myricetin-3-*O*-rhamnoside, as previously described(Mirali et al., 2014). Applying LC-MS/MS and comparing with standards, peak 13 (Rt=13.7) was identified as quercetin-3-*O*-rhamnoside.

*Anthocyanidins*. Glycones of malvidin, cyanidin and delphinidin were not detected in any of the samples.

*Flavan-3-ols*. The UV spectra contained a major absorbance peak at 274 nm for gallocatechin and its derivatives (Table 2). For catechin and its derivatives, the major absorbance peak was at 278-280 nm.(Abad-García et al., 2009) Gallocatechins eluted before catechins. Furthermore, the *cis* configuration (epi-) eluted later than the *trans* configuration; for example, (-)-epicatechin eluted 2 min after (+)-catechin. These configurations were monitored with the same MRM transition. Among the analyzed flavan-3-ols, only (-)-gallocatechin (peak 3), (+)-catechin (peak 7), and catechin-3-glucoside (peak 5)(Dueñas et al., 2003; Mirali et al., 2014) were detected in all four seed coat colours (Table 2; Figure 1).

*Proanthocyanidins*. Peak 4 was identified as procyanidin B1.(Mirali et al., 2014) Proanthocyanidins are the most abundant phenolic components in lentil.(Dueñas et al., 2002) As a result, SIR monitoring was applied to thoroughly investigate oligomers of proanthocyanidins (Table 3), most of which produced significant isotopic peaks that were not well resolved. Figure 2 shows the proanthocyanidin oligomer chromatograms overlaid each other in a gray seed coat/red cotyledon lentil sample (RIL group # 3), as an example. Chromatograms of the other seed coat/cotyledon combinations were similar. Peak 22 was characterized by a selected ion [M+H]^+^ at m/z 731.7 and a peak in the UV spectrum at 278 nm, and was identified as (epi)catechin – (epi)caetchin gallate (CC-gallate), a procyanidin dimer. Peaks 37 and 40 had an [M+H]^+^ at m/z 1155.9, corresponding to procyanidin tetramers with (epi)catechin monomer blocks (CCCC), eluting at 8.5 and 9.8 min, respectively. Peak 38 corresponded to a procyanidin pentamer with molecular ion at m/z 1444.1; this was the largest procyanidin found in all four seed coat background colours. Dimers to pentamers of prodelphinidins were also detected in all lentil samples (Table 3). Peaks 18 and 27 featured an [M+H]^+^ at m/z 595.6, which eluted at 3.8 and 6.6 min from the column, respectively, and correspond to prodelphinidin dimers with one (epi)catechin and one (epi)gallocatechin(Dueñas et al., 2003). Peaks 20, 33, 34, 16, 21, 24, and 19 were confirmed as prodelphinidin trimers with one ([M+H]^+^ at m/z 883.7), two ([M+H]^+^ at m/z 899.7), and three ([M+H]^+^ at m/z 915.6) (epi)gallocatechins. Prodelphinidin tetramers (peaks 29, 36, 25, 32, 17, 23, and 30) were identified by SIR with [M+H]^+^ at m/z 1171.8, 1187.8, and 1204.8. Peaks 35 and 39 correspond to prodelphinidin pentamers with one (epi)gallocatechin, while peaks 26 and 31 correspond to pentamers containing three (epi)gallocatechins building blocks.

Appendix Tables S1-S3 show the *P-*values from the ANOVA F-test for the effect of cotyledon and seed coat colour on mean area ratio per mg sample (MARS) of phenolic compounds at SPG, STH or the combination of these two locations. Resveratrol-3-*ß*-mono-*D*-glucoside and procyanidin B1 at STH location (Appendix Table S2) and vanillic acid-4-*ß*-*D*-glucoside and kaempferol-di-rutinoside in combination at two locations (Appendix Table S3) were not significantly different among seed coat colours, cotyledon colours or their interactions. Significant differences among MARS were observed for the remaining phenolic compounds.

Figures 3a-3c show mean comparisons for the main effect of seed coat colour at SPG, STH or the combination of both locations. For procyanidin B1 (at SPG location; Figure 3a) and GGGCC_I (at STH location; Figure 3b) , gray and green seed coat colour lentil samples have similar MARS values that are higher than brown and tan seed coat colour lentil samples. This trend observed for GGGC_I, GCCCC_I, and GGGCC_II (at both SPG and STH locations) and quercetin-3-*O*-rhamnoside, myricetin-3-*O*-rhamnoside and GCCCC_II (Figure 3c). The highest MARS values for flavan-3-ols including catechin (at both SPG and STH locations; Figures 3a,3b), gallocatechin (at STH location; Figure 3b), and catechin-3-glucoside (Figure 3c) are found in green seed coats. MARS values of resveratrol-3-*ß*-mono-*D*-glucoside (SPG location) demonstrate a different trend, where tan and green seed coat samples are similar but higher than brown and gray.

Figures 4a-4c compare the MARS values of phenolic compounds with respect to the interaction of seed coat and cotyledon colours. For gallocatechin (SPG location), green seed coats with either red and yellow cotyledon have significantly higher MARS values compared to brown, gray, and tan seed coats (Figure 4a). For GGC_I, GGC_II, GGG, GGCCs, GGGC_II, and GGGC_III, gray and green seed coats have significantly higher MARS values compared to brown and tan seed coats, especially for yellow cotyledons at SPG location. A similar trend is observed for luteolin-4’-*O*-glucoside, CC-gallate, CCCCs, CCCCC, GCs, GCCs, GGC_III, GGG, and GCCCs (Figure 4c). For kaempferol-3-*O*-robinoside-7-*O*-rhamnoside, green lentil seed coat with red cotyledon has a higher MARS value compared to the other samples, which have similar values.

To confirm if cotyledon colour influences the phenolic profile, dehulled seeds of two green seed coat/red cotyledon genotypes were compared to two green seed coat/yellow cotyledon genotypes (Figure 5). No proanthocyanidin oligomers were detected in any of the football fractions. Among the phenolic compounds detected in the cotyledons, the MARS values of kaempferol dirutinoside, catechin, and catechin-3-glucoside in red and yellow cotyledon colours were not significantly different. For vanillic acid-4-*ß*-*D*-glucoside and kaempferol-3-*O*-robinoside-7-*O*-rhamnoside, the MARS value for the red cotyledons was significantly higher than for the yellow cotyledons.

4. Discussion

Phenolic compounds are secondary metabolites with characteristics such as anti-oxidative properties that can play important roles in human health. In this study, we employed LC-MS to compare the profiles of more than 50 phenolic compounds in lentil seeds. Lentils with brown, gray, tan, and green seed coats with either red or yellow cotyledons were compared using seed samples grown from two locations. Vanillic acid-4-*ß*-*D*-glucoside, resveratrol-3-*ß*-mono-*D*-glucoside, luteolin-4'-*O*-glucoside, and several flavonols, flavan-3-ols, and proanthocyanidin oligomers were detected in all of our analyzed seed coat colours, similar to previous reports(Aguilera et al., 2010; Dueñas et al., 2002; Dueñas et al., 2003; López-Amorós et al., 2006; Xu and Chang, 2009, 2010).

An obvious trend was observed for luteolin-4’-*O*-glucoside (flavone sub-class), myricetin-3-*O*-rhmanoside and quercetin-3-*O*-rhmanoside (flavonol sub-class), and dimers, trimers, tetramers, and pentamers of proanthocyanidins, specifically that the MARS values were higher for gray (*Ggc tgc*) and green (*ggc tgc*) lentil seed coats compared to brown (*Ggc Tgc*) and tan (*ggc Tgc)* seed coats. Gray and green seed coats have the recessive *tgc* in common, while brown and tan seed coats both have the dominant allele (*Tgc*). This indicates that the production of some phenolic compounds, specifically proanthocyanidins, in lentil is controlled by the *tgc* seed coat colour gene. QTLs for tannins are located at the seed coat pattern gene *Z* (zonal) and seed coat colour *V* gene (violet factor) of common bean (*Phaseolus vulgaris*).(Caldas and Blair, 2009) *Z* and *V* genes of common bean map close to the phenylpropanoid pathway genes of 4-coumarate:CoA ligase (*4CL1*) and flavonoid-3’,5’-hydroxylase (*F3’5’H*), respectively.(Reinprecht et al., 2013) The *T* locus of soybean (*Glycine max*), which produces brown (*iRT*) vs. gray (*iRt*) seed coats, is associated with flavonoid-3’-hydroxylase (*F3’H*).(Toda et al., 2002) Anthocyanidins accumulate in the black (*iRT*) seed coat of soybean, but are undetectable in the brown (*irT*) seed coat. The genes affect anthocyanidins production are up-regulated in the black seeded soybean.(Kovinich et al., 2011) Proanthocyanidins are basically colourless, but secondary changes by polyphenol oxidase (PPO), for instance, might oxidize them and cause dark colours, i.e., a change from yellow to brown(Lepiniec et al., 2006; Marles et al., 2003). This might be similar to the origin of tan and brown colours in lentil. Oxidation of these phenolic compounds might have caused the reduction in the MARS values of the tan and brown genotypes. Therefore, *Tgc* might be linked to or associated with an oxidizing enzyme, while *tgc* could be a mutant form that results in less oxidation.

Although the types of phenolic compounds are consistent for different seed coat colours, the MARS value is affected by location and genotype. The random effect of location shows significant effect on some phenolic compounds (resveratrol-3-*ß*-mono-*D*-glucoside and several of the proanthocyanidins), while the rest were not affected by location (including vanillic acid-4-*ß*-*D*-glucoside, luteolin-4'-*O*-glucoside, catechin-3-glucoside, flavonols, and some proanthocyanidins). Within each location – and specifically SPG – the MARS values for gray and green seed coats are similar and higher than for tan and brown seed coats. The interaction of seed coat and cotyledon colour shows higher MARS values for green and gray seed coat colours, specifically for yellow cotyledons at SPG location. Vaillancourt *et al*. report a significant genotype × location interaction for total tannin content in lentil seed coat; however, the ranking of lentil lines is similar for different locations (Vaillancourt et al., 1986).

The main effect of seed coat colour or its interaction with cotyledon colour on the MARS value of catechin-3-glucoside, catechin, and gallocatechin indicate the highest values for the green seed coat. Catechin and its glycone were detected in both red and yellow dehulled samples. There may be an interaction from cotyledons for these flavan-3-ols that affects the MARS value of the analyzed whole seed.

Most of the phenolic compounds detected in the whole seed were not detected in the cotyledons, which is similar to previous reports of less diversity in phenolic compounds in cotyledons compared to seed coats.(Dueñas et al., 2002) Kaempferol dirutinoside, catechin, and catechin-3-glucoside were not significantly different between red and yellow cotyledons. However, red cotyledons had higher MARS values for vanillic acid-4-*ß*-*D*-glucoside and kaempferol-3-*O*-robinoside-7-*O*-rhamnoside compared to yellow cotyledons. The red cotyledon is controlled by a dominant allele of *Yc*, while the recessive form (*yc yc*) will produce the yellow cotyledon.(Slinkard, 1978) Cotyledon colour in lentil is associated with the amount of carotenoids.(Thomas, 2016) Investigation using a wide range of samples could help determine if compounds within the phenolic acid and flavonols sub-classes could affect cotyledon colour in lentil.

Higher antioxidant activity in lentil might be related to the presence of phenolic acids, flavonols, flavan-3-ols, and proanthocyanidins.(Fratianni et al., 2014; Xu and Chang, 2010; Zou et al., 2011) Lentil as part of the diet might contribute to the control of blood glucose levels and obesity because it contains flavonols.(Zhang et al., 2015) Considering the greater amounts of flavan-3-ols, proanthocyanidin oligomers, and some flavonols found in green and gray seed coats, these types might possess greater antioxidative and health promoting properties.

In conclusion, the MARS values of various phenolic compounds in lentil seeds varied with the different seed coat colours conferred by specific genotypes. Specifically, seed coats of lentil genotypes with the homozygous recessive *tgc* allele (green and gray seed coats) had higher amounts of flavan-3-ols, proanthocyanidins, and some flavonols. This suggests lentils featuring green and gray seed coats might be more promising as health-promoting foods.

Acknowledgment

**The authors** appreciate **financial assistance from the NSERC Industrial Research Chair Program and Saskatchewan Pulse Growers.** They also acknowledge additional support provided by the Pulse Research Crew at the Crop Development Centre, University of Saskatchewan.

Supporting Information description

Supplementary Tables S1, S2, and S3 are available in a separate file (Supp.docx).

References

Abad-García, B., Berrueta, L.A., Garmón-Lobato, S., Gallo, B., Vicente, F., (2009). A general analytical strategy for the characterization of phenolic compounds in fruit juices by high-performance liquid chromatography with diode array detection coupled to electrospray ionization and triple quadrupole mass spectrometry. J. Chromatog. A 1216, 5398-5415.

Aguilera, Y., Dueñas, M., Estrella, I., Hernández, T., Benitez, V., Esteban, R.M., Martín-Cabrejas, M.A., (2010). Evaluation of phenolic profile and antioxidant properties of pardina lentil as affected by industrial dehydration. J. Agric. Food Chem. 58(18), 10101-10108.

Andersen, Ø.M., Jordheim, M., (2010). Chemistry of flavonoid-based colors in plants, in: Mander, L.N., Liu, H.W. (Eds.), *Comprehensive Natural Products II: Chemistry and Biology*. Elsevier, Oxford, pp. 547–614.

Bartolomé, B., Estrella, I., Hernández, T., (1997). Changes in phenolic compounds in lentils (*Lens culinaris*) during germination and fermentation. Zlebensm Unters F. A. 205(4), 290-294.

Caldas, G.V., Blair, M.W., (2009). Inheritance of seed condensed tannins and their relationship with seed-coat color and pattern genes in common bean (*Phaseolus vulgaris* L.). Theoretical and Applied Genetics 119, 131–142.

Dueñas, M., Hernández, T., Estrella, I., (2002). Phenolic composition of the cotyledon and the seed coat of lentils (*Lens culinaris* L.). Eur. Food Res. Technol. 215(6), 478-483.

Dueñas, M., Sun, B., Hernández, T., Estrella, I., Spranger, M.I., (2003). Proanthocyanidin composition in the seed coat of lentils (*Lens culinaris* L.). J. Agric. Food Chem. 51(27), 7999-8004.

Fratianni, F., Cardinale, F., Cozzolino, A., Granese, T., Albanese, D., Matteo, M.D., Zaccardelli, M., Coppola, R., Nazzaro, F., (2014). Polyphenol composition and antioxidant activity of different grass pea (*Lathyrus sativus*), lentils (*Lens culinaris*), and chickpea (*Cicer arietinum*) ecotypes of the Campania region (Southern Italy). J. Funct. Food 7, 551–557.

Kovinich, N., Saleem, A., Arnason, J., Miki, B., (2011). Combined analysis of transcriptome and metabolite data reveals extensive differences between black and brown nearly-isogenic soybean (*Glycine max*) seed coats enabling the identification of pigment isogenes. BMC Genomics 12(1), 1-18.

Kuznetsova, A., Brockhoff, P., Christensen, R., (2016). lmerTest: Tests in Linear Mixed Effects Models. R package version 2.0-30.

Lepiniec, L., Debeaujon, I., Routaboul, J.-M., Baudry, A., Pourcel, L., Nesi, N., Caboche, M., (2006). Genetics and Biochemistry of Seed Flavonoids. Annu. Rev. Plant Biol. 57, 405–430.

López-Amorós, M.L., Hernández, T., Estrella, I., (2006). Effect of germination on legume phenolic compounds and their antioxidant activity. J. Food Comp. Anal. 19, 277–283.

Marles, M.A.S., Ray, H., Gruber, M.Y., (2003). New perspectives on proanthocyanidin biochemistry and molecular regulation. Phytochem. 64 367–383.

Mirali, M., Ambrose, S.J., Wood, S.A., Vandenberg, A., Purves, R.W., (2014). Development of a fast extraction method and optimization of liquid chromatography–mass spectrometry for the analysis of phenolic compounds in lentil seed coats. J. Chromatogr. B 969, 149–161.

R Core Team, (2016). R: A language and environment for statistical computing R foundation for statistical computing, Vienna, Austria.

Reinprecht, Y., Yadegari, Z., Perry, G.E., Siddiqua, M., Wright, L.C., McClean, P.E., Pauls, P.K., (2013). In silico comparison of genomic regions containing genes coding for enzymes and transcription factors for the phenylpropanoid pathway in Phaseolus vulgaris L. and Glycine max L. Merr. Front Plant Sci 4(317), 1-25.

Slinkard, A.E., (1978). Inheritance of cotyledon color in lentils. J Hered 69, 139-140.

Tar'an, B., Buchwaldt, L., Tullu, A., Banniza, S., Warkentin, T.D., Vandenberg, A., (2003). Using molecular markers to pyramid genes for resistance to ascochyta blight and anthracnose in lentil (*Lens culinaris* Medik). Euphytica 134, 223-230.

Thomas, T., (2016). Understanding the genetic basis of carotenoid concentration in lentil (*Lens culinaris* medik.) seeds, *Plant Sciences*. University of Saskatchewan.

Toda, K., Yang, D., Yamanaka, N., Watanabe, S., Harada, K., Takahashi, R., (2002). A single-base deletion in soybean flavonoid 3′-hydroxylase gene is associated with gray pubescence color. Plant Molecular Biology 50(2), 187-196.

Vaillancourt, R., Slinkard, A.E., Reichert, R.D., (1986). The inheritance of condensed tannin concentration in lentil. Can. J. Plant Sci. 66(2), 241-246.

Vandenberg, V., Slinkard, A.E., (1990). Genetics of seed coat color and pattern in lentil. J. Hered. 81(6), 484-488.

Vermeris, W., Nicholson, R., (2006). *Phenolic Compound Biochemistry*. Springer, Dordrecht, The Netherlands.

Xu, B., Chang, S.K.C., (2009). Phytochemical profiles and health-promoting effects of cool-season food legumes as influenced by thermal processing. J. Agric. Food Chem. 57(22), 10718-10731.

Xu, B., Chang, S.K.C., (2010). Phenolic substance characterization and chemical and cell-based antioxidant activities of 11 lentils grown in the northern United States. J. Agric. Food Chem. 58(3), 1509-1517.

Zhang, B., Deng, Z., Ramdath, D.D., Tang, Y., Chen, P.X., Liu, R., Liu, Q., Tsao, R., (2015). Phenolic profiles of 20 Canadian lentil cultivars and their contribution to antioxidant activity and inhibitory effects on a-glucosidase and pancreatic lipase. Food Chem. 172 862–872.

Zou, Y., Chang, S.K.C., Gu, Y., Qian, S.Y., (2011). Antioxidant activity and phenolic compositions of lentil (*Lens culinaris* var. Morton) extract and its fractions. J. Agric. Food Chem. 59(6), 2268-2276.

Figure captions

Figure 1. Typical chromatograms of a gray seed coat-red cotyledon lentil genotype obtained using LC and MRM mode. Peak numbers correspond to those given in Table 2, and the chromatographic conditions are as described in Table 2.

Figure 2. Typical chromatograms of a gray seed coat-red cotyledon lentil genotype obtained using LC and SIR mode. Peak numbers correspond to those given in Table 3, and chromatographic conditions are as described in Table 2.

Figure 3. Effect of four genetically distinct lentil seed coat colours (brown, gray, tan, and green) on mean area ratio per mg sample of phenolic compounds at (a) SPG location, (b) STH location, and (c) combination of two locations. Error bars are the standard errors of two cotyledon colours (3a and 3b) and two locations (3c) in three replicates. C and G stand for catechin/epicatechin and gallocatechin/epigallocatechin, respectively.

Figure 4. Interaction of cotyledon colour (red, yellow) and seed coat colour (brown, gray, tan, and green) on mean area ratio per mg sample of phenolic compounds at (a) SPG location, (b) STH location, and (c) combination of two locations. Error bars are the standard errors for three replicates (4a and 4b) or two locations with three replicates (4c). Kaemp-robin-rhman, C, and G stand for kaempferol-3-*O*-robinoside-7-*O*-rhamnoside, catechin/epicatechin and gallocatechin/epigallocatechin, respectively.

Figure 5. Mean area ratio of different phenolic compounds per mg of red and yellow cotyledons. Error bars are the standard errors of two genotypes with three replicates.
